# The Characteristics and Prognostic Effect of E-Cadherin Expression in Colorectal Signet Ring Cell Carcinoma

**DOI:** 10.1371/journal.pone.0160527

**Published:** 2016-08-10

**Authors:** Renjie Wang, Xiaoji Ma, Yaqi Li, Yiping He, Dan Huang, Sanjun Cai, Junjie Peng

**Affiliations:** 1 Department of Colorectal Surgery, Fudan University Shanghai Cancer Center, Shanghai, P. R. China; 2 Department of Oncology, Shanghai Medical College, Fudan University, Shanghai, P. R. China; 3 Department of Endoscopy, Fudan University Shanghai Cancer Center, Shanghai, P. R. China; 4 Department of Pathology, Fudan University Shanghai Cancer Center, Shanghai, P. R. China; University of Navarra, SPAIN

## Abstract

**Purpose:**

Signet ring cell carcinoma (SRCC) is rare. The aim of this study is to understand the clinicopathological features and identify the possible prognostic factors in colorectal SRCC.

**Methods:**

Patients with SRCC who underwent primary lesion resection at Fudan University Shanghai Cancer Center from September 2008 to July 2014 were retrospectively analyzed. Patient’s gender, age, tumor location, depth of invasion, lymph node metastasis, synchronous distant metastasis, perineural invasion, lymphovascular invasion, and E-cadherin expression were studied with prognosis, and the correlation between E-cadherin expression and clinicopathological features were analyzed. All clinicopathological and molecular factors were put into multivariate analysis using Cox proportional hazards model for detecting independent prognostic factors.

**Results:**

59 patients accounting for 0.89% of total colorectal cancer patients met the criteria and were enrolled in the study. The median survival time is 28.9 months, and the 3-year survival rate is 62.7%. SRCC were seen more common in young male patients. Advanced stage was more common in SRCC, 58 (98.3%) patients had T3/T4 lesions, 52 (88.1%) patients had lymph node metastasis, and 14 (23.7%) patients had distant metastasis. Distant metastases were seen more common in peritoneal cavity. Distant metastasis (HR = 4.194, 95% CI: 1.297–13.567), lymphovascular invasion (HR = 2.888, 95% CI: 1.115–7.483), and E-cadherin expression (HR = 0.272, 95% CI: 0.096–0.768) were independent predictors for survival.

**Conclusions:**

SRCC is a rare subtype of colorectal cancer with poor prognosis. Distant metastasis, lymphovascular invasion, and E-cadherin expression can predict prognosis of colorectal SRCCs independently. More precise therapy and more close surveillance are needed for these patients.

## Introduction

Signet ring cell carcinoma (SRCC) is a rare type of adenocarcinoma, which is characterized by specific morphologic appearance of abundant intracytoplasmic mucin pushing nucleus to periphery and giving it a signet ring-like appearance. The World Health Organization classification of tumors has a clear definition for diagnosis of this subtype: SRCC is defined as presence of more than 50% of signet ring cells[1]. SRCCs are most commonly seen in stomach (95%) and occasionally found in colon, rectum, ovary, peritoneum, and gallbladder. SRCC in colorectum is rare, and the first case was reported by Laufman and Saphir in 1951[2]. Because of its rarity, the characteristics of this subtype are seldom described in detail. Till now, most of the reported articles are case reports or small series, and few investigations have explained the clinical behavior of this subtype. At present, it is generally acknowledged that colorectal SRCC has poorer prognosis and treatment response than conventional adenocarcinoma. The reported 5-year survival rate varied from 0% to 31%, and the median survival time was 15–45 months[3–11]. In fact, most of patients with colorectal SRCC were already in stage III or IV at diagnosis, and the reported percentage of patients diagnosed at early stage was only around 5%[11]. So clarifying SRCC’s clinicopathological features and identifying possible prognostic factors are essential in order to improve early detection, treatment and surveillance for this distinctive phenotype. E-cadherin is a cadherin family member and a calcium-dependent cell-to-cell adhesion molecule found mainly in epithelial tissue. It is thought to implicate embryogenesis, cellular migration, and cellular differentiation or dedifferentiation[12]. Many investigators have suggested the suppressor role of E-cadherin in tumor invasion[13]. Loss or diminished E-cadherin expression has been demonstrated in many epithelial cancers[14–16]. However, its function in colorectal SRCC has not been studied yet. Hence, we conducted a retrospective study on colorectal SRCC patients in our hospital to elucidate the clinicopathological features of colorectal SRCC and to identify the clinical correlation and possible prognostic factors including biological characteristics of E-cadherin in colorectal SRCC.

## Materials and Methods

### Patients

Patients with SRCC who underwent primary lesion resection at Fudan University Shanghai Cancer Center from September 2008 to July 2014 were reviewed in this study. Written informed consent was obtained from all study participants adhering to the local ethical guidelines prior to specimen collection. The study protocol and consent procedure were approved by the Ethics Committee of Fudan University Shanghai Cancer Center. All the patients met the criteria as follows: (1) the presence of signet ring cells in > 50% of total tumor cells; (2) having complete medical records including demographic information, clinical and pathological data, operation notes, and follow-up results; (3) having at least 6 months follow-up time after the operation. Patients who (1) had preoperative chemoradiotherapy before operation; (2) had other histological types including adenosquamous carcinoma, squamous carcinoma, neuroendocrine tumor, clear-cell carcinoma, spindle cell carcinoma, and anaplastic carcinoma; (3) had incomplete paraffin block were all excluded from the study. The cancer staging was based on the American Joint Committee on Cancer 7th edition. Patient’s gender, age, tumor location, depth of invasion, lymph node metastasis, distant metastasis, perineural invasion, lymphovascular invasion, and E-cadherin expression were analyzed in this study.

### Immunohistochemial staining and interpretation

Tissues were embedded in paraffin. Sections were cut in 4 μm, dewaxed in xylene, and rehydrated in decreasing concentrations of ethanol. Prior to staining, sections were subjected to endogenous peroxidase blocking in 1% of H_2_O_2_ solution for 20 min and then to antigen retrieval treatment in 10 mM citrate buffer (pH 6.0) and in 95°C water bath for 40 min. Serum blocking was performed using 5% BSA for 20 min. Primary antibodies against E-cadherin (Clone NCH-38, Dako, Glostrup, Denmark) were incubated overnight at 4°C at 1:250 dilution. After washing, sections were incubated with mouse and horseradish-peroxidase-labeled streptavidin, respectively (1:200 dilution each). Diaminobenzidine hydrochloride (DAB) was used as the chromogen. Finally, they were counterstained with hematoxylin.

**Fig 1:**
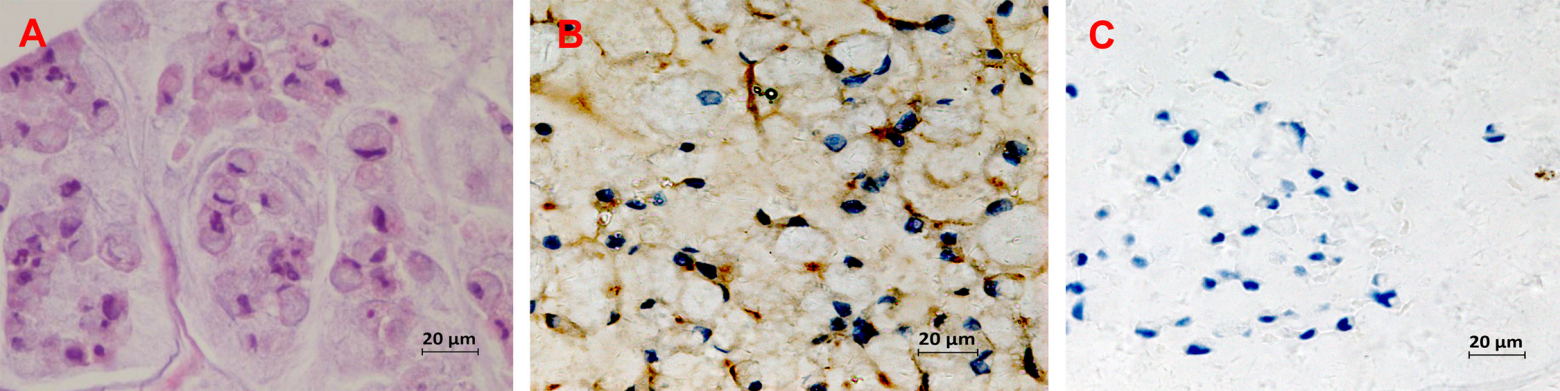
**Expression of E-cadherin in colorectal SRCC tissues** (A) Hematoxylin and eosin staining; Examples of Immunohistochemical staining of E-cadherin at positive expression (B) and negative expression (C).

We randomly selected 10 fields at a high power magnification for evaluation. Generally, we applied a two grade scoring (negative or positive) for the expression of E-cadherin. We focused on the membranous staining pattern, and cytoplasmic or nuclear staining was not noted. Staining was scored as positive when immunoreactivity in the tumor region showed a similar membranous staining to its normal counterpart in more than 25% of the cells. Absence of membranous staining or positive immunoreactivity in less than 25% of the cells was graded as negative (Fig 1).

### Statistical analysis

The clinicopathological features and E-cadherin expression status were analyzed using survival curves. Survival curves were generated by the Kaplan-Meier method, and univariate survival distributions were compared using log-rank test. The χ^2^ test was used to evaluate the statistical significance of the association of E-cadherin expression with patients’ clinicopathological parameters. All clinicopathological and molecular factors were included in multivariate analysis by using Cox proportional hazards model. The 2-tailed *p* value for significance was established at 0.05. All statistical analysis was performed by SPSS, version 19.0.0 (SPSS, Inc., Chicago, IL, USA).

## Results

### Clinicopathological characteristics

A total of 59 patients (38 men and 21 women) with colorectal SRCC met the criteria and were enrolled in the study, which accounts for 0.89% (59/6625) of the total resected colorectal cancer in our center. The median age of the diagnosis of SRCC was 46 y (range, 24–82 y). 58 (98.3%) patients had T3/T4 lesions, 52 (88.1%) patients had lymph node metastasis, and 14 (23.7%) patients had distant metastases. Among 14 patients with synchronous metastases, 5 (35.7%) patients had liver metastases only, 8 (57.1%) patients had diffusive intro-abdominal peritoneal metastases, and 1 (7.2%) patient had ovarian metastasis. With a median follow-up time of 19 months (range, 3.1–49.2 months), the median overall survival time is 28.9 months for all patients; the 3-year overall survival rate is 62.7%. The detailed clinicopathological characteristics are shown in Table 1.

**Table 1:** Patients' clinicopathologic features and survival (Kaplan-Meier).

Variables	N(%)	3-year survival rate(%)	*P*
Age			0.871
<40	16(27.1)	43.0	
≥40	43(72.9)	50.0	
Gender			0.702
Male	38(64.4)	49.4	
Female	21(35.6)	53.6	
Location*			0.475
Distal	45(76.3)	42.8	
Proximal	14(23.7)	70.1	
Invasive depth			0.896
T1-T2	1(1.7)	100	
T3-T4	58(98.3)	49.7	
Lymph nodes metastasis			0.691
Yes	52(88.1)	47.4	
No	7(11.9)	66.7	
Distant metastasis			**0.005**
Yes	14(23.7)	16.1	
No	45(76.3)	60.8	
Perineural invasion			0.475
Yes	43(72.9)	45.2	
No	16(27.1)	73.1	
Lymphovascular invasion			**0.049**
Yes	26(44.1)	33.3	
No	33(55.9)	63.5	
Ecadherin expression			**0.010**
(+)	23(39.0)	75.0	
(-)	36(61.0)	32.9	

*Distal location includes descending colon, sigmoid colon and rectum; Proximal location includes cecum,ascending colon and transverse colon; *P* is calculated using unadjusted log-rank test.

By immunohistochemical staining, 23 (39.0%) patients were scored as E-cadherin positive, while 36 (61.0%) patients were scored as E-cadherin negative. E-cadherin expression was not related to patients’ clinicopathological features (Table 2).

**Table 2:** Patients’ clinicopathologic features and E-cadherin expression.

Variables	N	E-cadherin expression		*P*
		negative (n = 36)	positive (n = 23)	
Age				0.371
<40	16	8	8	
≥40	43	28	15	
Gender				0.405
Male	38	25	13	
Female	21	11	10	
Location*				0.734
Distal	45	28	17	
Proximal	14	8	6	
Invasive depth				1.000
T1-T2	1	1	0	
T3-T4	58	35	23	
Lymph nodes metastasis				0.899
Yes	52	32	20	
No	7	4	3	
Distant metastasis				0.532
Yes	14	10	4	
No	45	26	19	
Perineural invasion				0.647
Yes	43	27	16	
No	16	9	7	
Lymphovascular invasion				0.942
Yes	26	16	10	
No	33	20	13	

*Distal location includes descending colon, sigmoid colon and rectum; Proximal location includes cecum,ascending colon and transverse colon; *P* is based on χ^2^ test/Fisher’s exact test.

### Survival analyses

**Fig 2:**
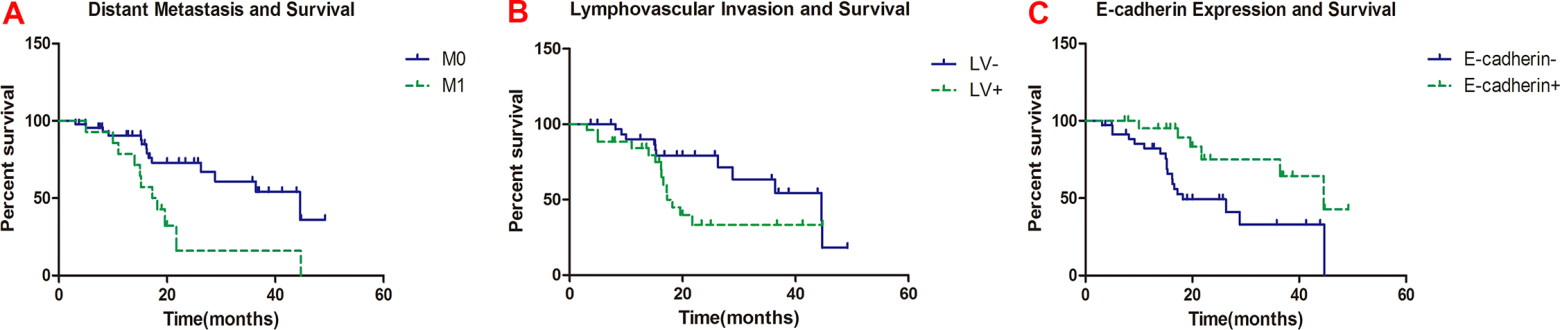
Patient’s clinicopathologic features/E-cadherin expression and survival. (A) Distant metastasis and survival;(B) Lymhovascular invasion and survival; (C) E-cadherin expression and survival.

The clinicopathological characteristics and E-cadherin expression were included in univariate analysis. Distant metastasis (*p* = 0.005), lymphovascular invasion (*p* = 0.049), and E-cadherin expression (*p* = 0.010) were found related to patients’ overall survival (Table 1, Fig 2). All clinicopathological and molecular variables were included in multivariate analysis. The result showed that distant metastasis (HR = 4.194, 95% CI: 1.297–13.567), lymphovascular invasion (HR = 2.888, 95% CI: 1.115–7.483), and E-cadherin expression (HR = 0.272, 95% CI: 0.096–0.768) were independent prognostic factors for patients’ overall survival (Table 3).

**Table 3:** Multivariate analysis of survival in colorectal SRCC.

Factor	HR	*P*	95% CI	
			Lower	Upper
Gender	1.547	0.439	0.512	4.675
Age	1.506	0.509	0.447	5.077
Location	0.657	0.508	0.189	2.281
Lymph node metastasis	1.289	0.789	0.201	8.246
Distant metastasis	4.194	**0.017**	1.297	13.567
Perineural invasion	1.695	0.411	0.482	5.962
Lymphovascular invasion	2.888	**0.029**	1.115	7.483
E-cadherin expression	0.272	**0.014**	0.096	0.768

Abbreviation: HR,harzard ratio; CI, confidence interval; *P* is based on Cox regression test.

## Discussion

Colorectal SRCC is a rare disease, which comprises approximately 0.1–2.6% of all colorectal malignancies[7–10]. In our surgical series, SRCC comprised 0.89% (59/6625) of the resected colorectal cancer, being 0.90% (27/3009) of colon cancer and 0.88% (32/3616) of rectal cancer, which was comparable to the reported incidence in Western countries.

SRCC was reported more prevalent in patients at younger age (≤ 40 y) than conventional colorectal adenocarcinoma[4,6,17,18]. In our study, the median age of colorectal SRCC was 46 years old, and 66% of patients were younger than 40 years old, which is obviously higher than conventional colorectal adenocarcinoma. Some studies found that colorectal SRCC is slightly more prevalent in women than in men[7,19], while others demonstrated a clear male predominance[9,20–23]. In this study, we observed more male SRCC patients than female, with a ratio of 1.5: 1. More reported colorectal SRCC cases are localized exclusively in the rectum[8,10,17,19] whereas some studies reported a right-sided predominance[9,23]. In our study, more SRCCs were located in rectum, but all sites were involved and the distribution of cases between colon and rectum was similar.

Advanced stage is more common in SRCC, and most patients develop lymph node metastasis or distant metastasis when diagnosed[7–10]. In our study, almost all cases (98.2%) were T3 and T4, and only 1 case was T2. SRCCs are considered to have early invasion; possibly, this is because early SRCCs are usually asymptomatic and are normally a tumor arising in flat colonic mucosa but not following the adenocarcinoma sequence[24]. Thus, it is difficult to find SRCC by routine colonoscopy at early stage and hard to obtain a definitive diagnosis through biopsy. In our series, most cases (89%) had lymph node metastasis, and among them, 72.7% cases had 4 or more than 4 lymph node metastasis. This metastatic rate was consistent with previous studies[7–10]. Loss of early detection could partly explain the high rate of lymph node metastasis, and other reasons can be explained by SRCCs’ unique metastatic routes. In our study, 14 patients had distant metastasis. Among them, 5 (35.7%) patients had liver metastases, 8 (57.1%) patients had diffusive intro-abdominal peritoneal metastases, and 1 (7.1%) patient had ovarian metastasis. We also found distant metastasis was a significant and independent predictor for prognosis; median survival for patients with distant metastasis was only 17.3 months compared with patients without distant metastasis (44.6 months). As we have known, in conventional adenocarcinoma, liver metastasis and lung metastasis accounts for nearly 90% of total distant metastasis. However, in SRCC, distant metastasis in peritoneal cavity is more frequent. In gastric cancer, signet ring cell carcinoma also tends to develop peritoneal metastasis than other types more frequently[25]. Likewise, Pende et al. found that conventional colorectal adenocarcinoma containing signet ring cell component had a higher rate of peritoneal metastasis[26]. So, high incidence of peritoneal metastasis and a relatively low incidence of liver and lung metastasis are characteristic features of SRCC. Since liver/lung metastasis may still have chances for radical resection, whereas diffusive peritoneal metastasis means loss of surgical chances, this may lead to poor prognosis for SRCC.

In conventional adenocarcinoma, the percentage for lymphovascular invasion is around 20%, in our study, 26 (44.1%) patients had lymphovascular invasion, the median survival for LV+ patients were 17.3 months compared with LV- patients (44.6 months), and lymphovascular invasion was found to be an independent predictor for prognosis. So, the percentage of lymphovascular invasion in SRCC is higher than conventional adenocarcinoma. Some articles have already reported that lymphovascular invasion was a prognostic variable in conventional adenocarcinoma[27,28]. Since lymphovascular invasion means invasion of tumor cells into lymph or blood vessels, which is important in metastatic process, and SRCC has a high rate of metastasis, we have reasons to believe that lymphovascular invasion can be a good factor for predicting prognosis in colorectal SRCC.

E-cadherin plays a crucial role in cell-to-cell adhesion and maintaining epithelial morphology. Reduced expression of E-cadherin due to aberrant hypermethylation is important for metastases in multiple cancers. In conventional colorectal adenocarcinoma, the rate of loss of E-cadherin expression was reported between 13.0%-41.7% and down-regulated E-cadherin expression predicted worse prognosis[29–32]. In our study, the rate of loss of E-cadherin expression (61.0%) was higher than that reported in conventional adenocarcinoma. Survival analysis found the median survival time for E-cadherin- patients were 18.2 months compared with 44.6 months in E-cadherin+ patients. Multivariate analysis confirmed that loss of E-cadherin expression was a significant and independent predictor for poor prognosis. E-cadherin, a 120-kd transmembrane glycoprotein, is a calcium-dependent cell adhesion molecule involved in inducing and maintaining the epithelial cell polarity[33,34]. The cytoplasmic tail of E-cadherin binds to catenins and the formation of E-cadherin and β-catenin complexes is critical for epithelial cell functions and tissue integrity[35,36]. Loss of E-cadherin/catenin complex has been regarded as the cause of loss of epithelial differentiation or architecture and acquisition of a motile and invasive phenotype[37], which may allow certain cancer cells detached from the surrounding structure and become more infiltrative and metastasizing. Moreover, Wnt signaling pathway is the cause of approximately 93% of colorectal cancer[38] and β-catenin is a critical component of the Wnt signal pathway. The upregulation of Wnt signal pathway induces nuclear translocation of β-catenin and aberrant target gene expression[39,40]. Downregulation of E-cadherin may affect the decreased membrane expression of β-catenin and resulting in its nuclear shifting which may also enhance Wnt signal pathway and give rise to aberrant proliferation.

In our series, the overall 3-year survival rate was 62.7%, with a median survival of 28.9 months, which was significantly worse in comparison to the rates of conventional adenocarcinoma and was in accordance with previous studies[7–10]. Otherwise, we found that traditional prognostic variables such as depth of invasion and lymph node metastasis were not related to prognosis, this may partly because most cases were T3-T4 and N+, and there were too few early cases.

There are still limitations in our study. This is a retrospective study and some patients were excluded because of loss of follow-up or complete records. The adjuvant chemotherapy or radiation therapy was not completely disciplined because of the long study periods. And the follow-up periods were still not long enough for drawing a popular 5-year survival. Despite these limitations, our study represented one of the largest retrospective studies of colorectal SRCC.

In conclusion, colorectal SRCC is a rare type of colorectal cancer and is more commonly seen in young male patients. SRCC has poorer prognosis compared with conventional adenocarcinoma. Loss of E-cadherin expression was more common in colorectal SRCC and was one of important prognostic factors for predicting patients’ overall survival. Further studies were required to clarify the value and mechanism of E-cadherin expression in colorectal SRCC.

## Supporting Information

S1 File Dataset for SRCC study. (XLSX)
